# Mechanical properties of a bioabsorbable magnesium interference screw for anterior cruciate ligament reconstruction in various testing bone materials

**DOI:** 10.1038/s41598-023-39513-8

**Published:** 2023-07-31

**Authors:** Nad Siroros, Ricarda Merfort, Yu Liu, Maximilian Praster, Filippo Migliorini, Nicola Maffulli, Roman Michalik, Frank Hildebrand, Jörg Eschweiler

**Affiliations:** 1 Department of Orthopaedics, Trauma, and Reconstructive Surgery, University Hospital RWTH Aachen, Pauwelsstraße 30, 52074 Aachen, Germany; 2 Department of Orthopaedic and Trauma Surgery, Academic Hospital of Bolzano (SABES-ASDAA), 39100 Bolzano, Italy; 3 Department of Medicine, Surgery and Dentistry, University of Salerno, 84081 Baronissi, Italy; 4 School of Pharmacy and Bioengineering, Keele University Faculty of Medicine, Stoke on Trent, ST4 7QB England; 5 Barts and the London School of Medicine and Dentistry, Centre for Sports and Exercise Medicine, Mile End Hospital, Queen Mary University of London, London, E1 4DG England

**Keywords:** Experimental models of disease, Preclinical research, Translational research

## Abstract

Tears of the anterior cruciate ligament (ACL) negatively impact the stability and kinematics of the knee. Interference screws (ISs) are used for graft fixation in ACL reconstruction and provide sufficient fixation strength to withstand the patients' activities during the graft-to-bone integration process. Magnesium is a novel material used to manufacture IS given its strength and bioabsorbability. In previous studies, the selected magnesium IS design showed a better fixation performance in comparison to the conventional IS design due to its shape design and surface condition. In this study, bioabsorbable magnesium ISs were tested for their insertion (insertion torque and a number of turns to implement the IS) and fixation performance (pull-out and dynamic test). To obtain a reliable initial assessment of IS performance, ISs were implanted in 15 per cubic foot (PCF) Sawbones polyurethane foam blocks, Sawbones biomechanical tibia models with 17 PCF foam cores, and human cadaveric tibiae. Porcine tendons were used in the foam block pull-out test, and nylon ropes were used in all other test setups to prevent influences of the ligament graft material itself. In the pull-out test, the graft was subjected to tensile stress at a rate of 6 mm/min. For the dynamic test, 1000 cycles between 0 and 200 N were performed, followed by a final pull-out test. After each test, the tunnel widening pattern was observed by measuring the aspect ratio of the tunnel at the insertion site. The insertion torque lies within the normal insertion torque of the ISs as well as the average ligament tension before the insertion. In the foam block setup, the nylon rope showed a higher pull-out force than the porcine tendon. The comparison of each setup using nylon rope for both pull-out and pull-out after the dynamic test showed no significant difference between the foam block and cadaver setup. However, all tibia model setup shows unexpectedly high pull-out force due to the influence of its cortical layer. There were no statistically significant differences in tunnel widening between foam block-porcine tendon and foam block-nylon rope constructs. The pull-out resistance of magnesium ISs falls within the typical ACL tension range during daily activities. Even though the test results of the magnesium ISs are different in each bone material, the magnesium IS shows adequate fixation ability and workability during insertion without material failure.

## Introduction

The anterior cruciate ligament (ACL) influences the stability and kinematics of the knee joint^1^. Tears of the ACL are common^2^ and can lead to additional pathological changes in and around the knee joint^3^. Intra-articular reconstruction of the ACL with fixation of the graft by interference screw (IS) is routinely performed^4–6^ with a success rate of 75–95%^3,7^. In Germany, 81% of surgeons preferred fixation with ISs, preferably with a bioabsorbable IS (60%), especially for the tibia^8^, where failure mainly occurs^9^. Permanent metal (e.g. titanium) and polymeric ISs have been used, with no significant differences in clinical outcome^10,11^. Metallic ISs may cause graft damage and induce tunnel widening^3^. The polymer material is weaker than metal, and screw fracture during their insertion can occur^3,12^.

Magnesium, a light metal, is an excellent alternative to standard metallic materials and several characteristics have been studied^3,13,14^. Its bioabsorbability and biocompatibility in combination with high strength are advantageous compared to other materials^15^. In addition, magnesium alloys have been used as an alternative biodegradable material for fracture implants because their mechanical properties (Young's modulus, Poisson ratio) are similar to those of human cortical bone^16^. Magnesium shows bone-comparable material properties and a controlled absorption rate, is radiotransparent and produces low MRI artifact, with adequate graft fixation strength and biodegradability after implantation in humans. In addition to material selection, IS design parameters such as the slope profile and surface condition also influence IS performance. In a previous study, the magnesium IS design selected for this study resulted in a promising outcome in comparison to the conventional IS design, given its slope profile, and surface condition^14^. The mechanical performance of magnesium IS can be evaluated considering two parameters: insertion torque and fixation strength. The performance testing method for ISs approximated the guideline for the in vitro testing of the cruciate ligaments and ligament reconstruction^17^, and was adapted from the standard specification and test methods for metallic medical bone screws (ASTM F543). Fixation strength is determined via a pull-out test (load-to-failure test) and by a dynamic test. The pull-out test can be performed with a pulling rate ranging from 5 to 1000 mm/min^17^. The dynamic test is performed through 1,000 cycles which can evaluate the initial response of the IS-graft construct^17^. The ligament should be preconditioned in a low cyclic-low load manner, for example, with 20 cycles ranging between 0 and 50 N^18^. Three materials are commonly employed for IS testing: polyurethane foam blocks, a Sawbones tibia model, and human cadaveric knees^3,4,19–22^. Because of variations in the tests in terms of screw design, bone material, and test setup, it is difficult to compare all of the results of one test to another.

The focus of this biomechanical investigation is on tibia fixation using bioabsorbable magnesium ISs. The insertion and fixation performance of the IS is determined by the insertion torque, number of attempts used to turn the IS to complete the insertion (no. of turns), pull-out test, and dynamic test (with pull-out test afterward). As stated, there are three commonly used bone materials in IS testing. However, the morphological structure of the three materials is different, and there is no evidence that the results among the three materials are directly comparable. The use of the three bone materials will help to determine the performance of the ISs according to the variation in bone material characteristics. In addition, it should reflect the appropriate utilization of the bone material in a specific experimental setup. Therefore, this study aims to evaluate and compare the in vitro insertion and fixation performance of the IS in human cadaveric tibiae, polyurethane foam blocks, and artificial tibiae models. The hypothesis of this study is the IS performs similarly across all bone materials, the insertion torque stays within 3 Nm, and each IS functions properly during tests without screw failure in all test setups.

## Methods

The experiment assessed the insertion (insertion test) and fixation performance (pull-out and dynamic tests) of the IS in four configurations of different “bone material-ligament material” combinations (test setup) shown in Table 1. In each test, the insertion test was followed by a pull-out or a dynamic test. Furthermore, after the dynamic test, a pull-out test was performed. The pull-out force was investigated using a distance-controlled load-to-failure approach. Furthermore, the initial ligament tension, insertion torque, the number of insertion turns, and tunnel widening pattern were recorded and analyzed.

**Table 1:** Summary of the test setup.

Test setup	Pull-out test	Dynamic test
Foam block with porcine tendon	n = 5	–
Foam block with nylon rope	n = 5	n = 5
Tibia model with nylon rope	n = 5	n = 5
Cadaveric tibia with nylon rope	n = 5	n = 6

### Materials and pre-test preparation

**Figure 1:**
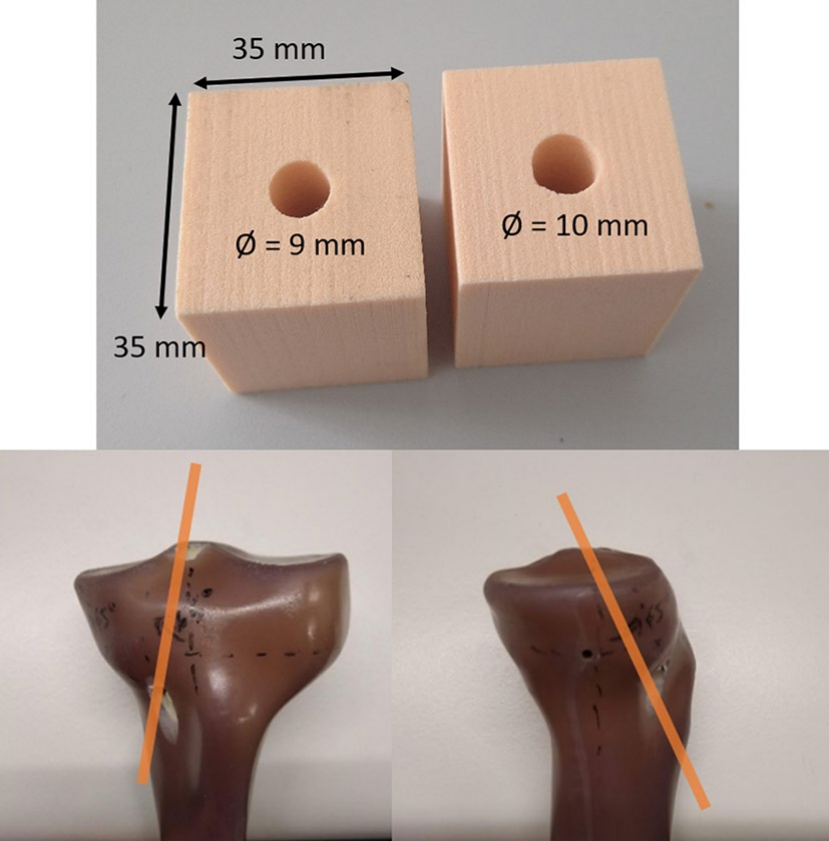
Examples of the bone block with 9 mm and 10 mm diameter tunnel (top) and tibia model with a tunnel (bottom).

The magnesium IS used in this study measured 9 mm in diameter and 30 mm in length with 2.5 mm pitch and 0.825 mm pitch depth. The pitch and pitch depth were measured using a Vernier calliper (ACCUD Co., Ltd., SuZhou, China). Two different ligament materials were used as ACL grafts: porcine flexor tendons (average diameter 7.7 ± 0.6 mm) and 9 mm in diameter nylon ropes (Kanirope GmbH, Dortmund, Germany). The fresh-frozen porcine legs were purchased from a local butcher; therefore, ethical approval is not required. The porcine legs were carefully defrosted overnight and the tendon harvested as previously described^23^. The dissected tendons were stored in phosphate-buffered saline (PBS). Moreover, the 15 per cubic foot (PCF) solid rigid polyurethane foam block (Sawbones, Pacific Research Laboratories, WA, USA) were prepared in size 35 mm × 35 mm × 40 mm (length x width x height) with a drilled 9 mm tunnel (n = 5) and 10 mm tunnel (n = 10), for test with porcine tendon and nylon rope, respectively (Fig. 1 top). The tunnel for the tibia model (Tibia 4th Generation 17 PCF large size, Sawbones, Pacific Research Laboratories, WA, USA) (n = 10) was drilled using a CNC machine (DMG MORI Global Marketing GmbH, Wernau, Germany) at 65° in the sagittal and coronal planes as shown in Fig. 1 (bottom). All cadaveric knees (n = 11) were explanted from three females and eight males, ages 76–95 (average of 83.6 ± 6.6 years old) provided by the Institute of Molecular and Cellular Anatomy (MOCA), Uniklinik RWTH Aachen. The cadavers were thawed overnight to room temperature and dissected before testing. A consultant orthopaedic surgeon visually inspected the tibia to determine whether it could be used for the experiment. The tibia models and cadavers were fixed to the machine using polyurethane foam (Euro-Leder GmbH & Co.KG, Georgsmarienhütte, Germany).

### Insertion test

**Figure 2:**
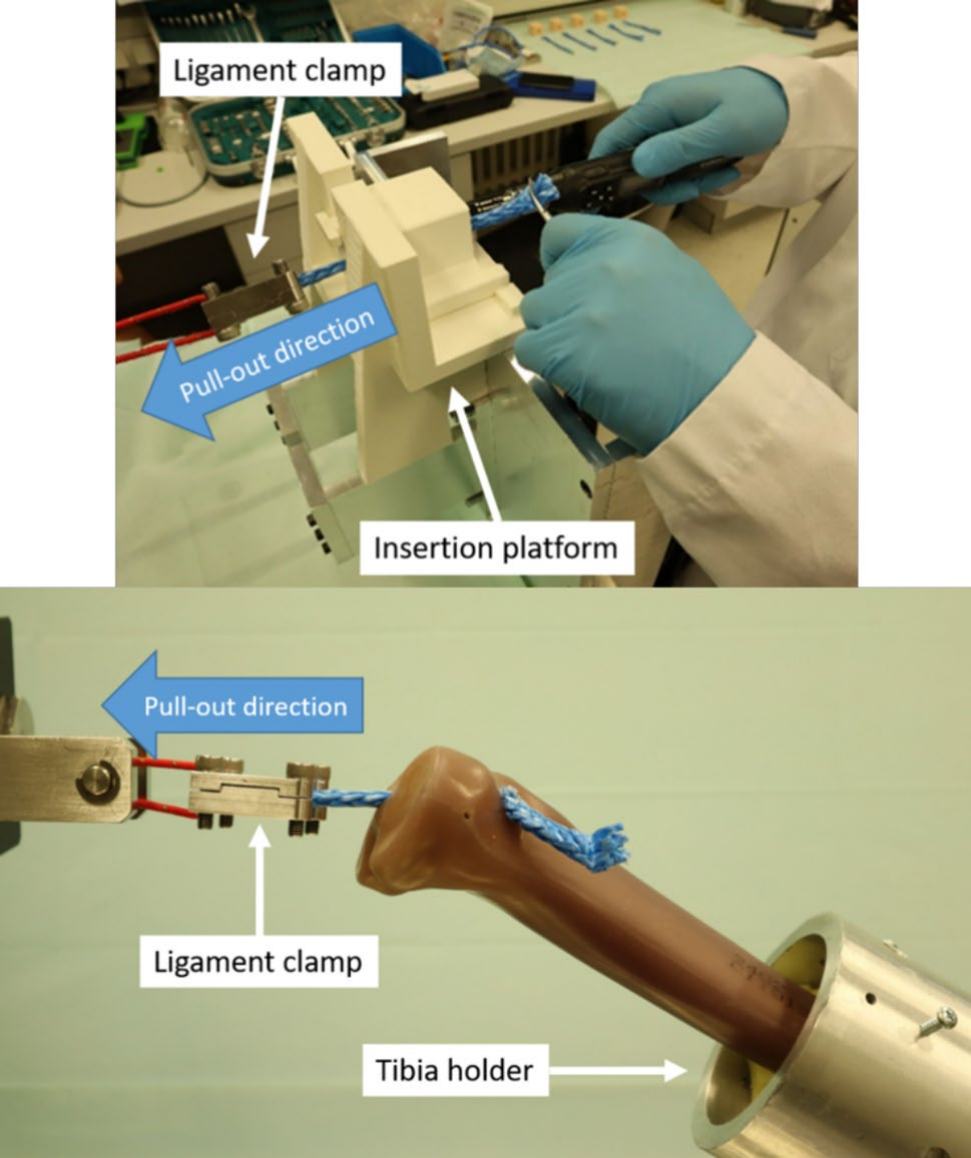
Screw insertion using foam block (top), and tibia model after screw insertion (bottom).

The process of screw insertion into the foam block is shown in Fig. 2 (top). The block was brought into the 3D-printed insertion platform where it was fixed. The ligament material (porcine tendon and nylon rope) was connected to the 2.5 kN load cell of the testing machine (DYNA-MESS Prüfsysteme GmbH, Germany) using a specially designed ligament clamp. The insertion tunnel and the ligament material were aligned coaxially to the measuring axis of the load cell. The ligament material was pulled through the tunnel. Before the IS insertion, tension was applied to the ligament by the consultant surgeon, and the tension force was recorded in Newton [N]. The insertion torque was measured using a digital torque screwdriver (TSD-400, Electromatic Equip't Co., Inc., NY, USA). Since the torque increases once the IS progresses into the tunnel, the final torque is also the maximum torque (referred to as the insertion torque). The average insertion torque of each setup was then analyzed. Furthermore, to successfully insert the IS, the surgeon must progress the IS into the tunnel by turning the screwdriver until the IS is completely inserted in the tunnel. The number of attempts trying to turn the IS is referred to as “the number of turns”^4^. Hence, one complete rotation of the IS may consist of multiple numbers of turns. Therefore, in the insertion test, the number of turns and the insertion torque [Nm] were recorded. For the screw insertion setup of the artificial tibia model, the model was fixed with a tibia holder (Fig. 2 (bottom)) in which the tunnel is also aligned coaxially to the load. The tibia was not pre-drilled for the cadaveric tibia screw insertion setup. Therefore, it was first fixed into the tibia holder, and the tunnel was drilled using standard instruments for ACL reconstruction (Arthrex GmbH, Munich, Germany).

### Pull-out test and dynamic test

**Figure 3:**
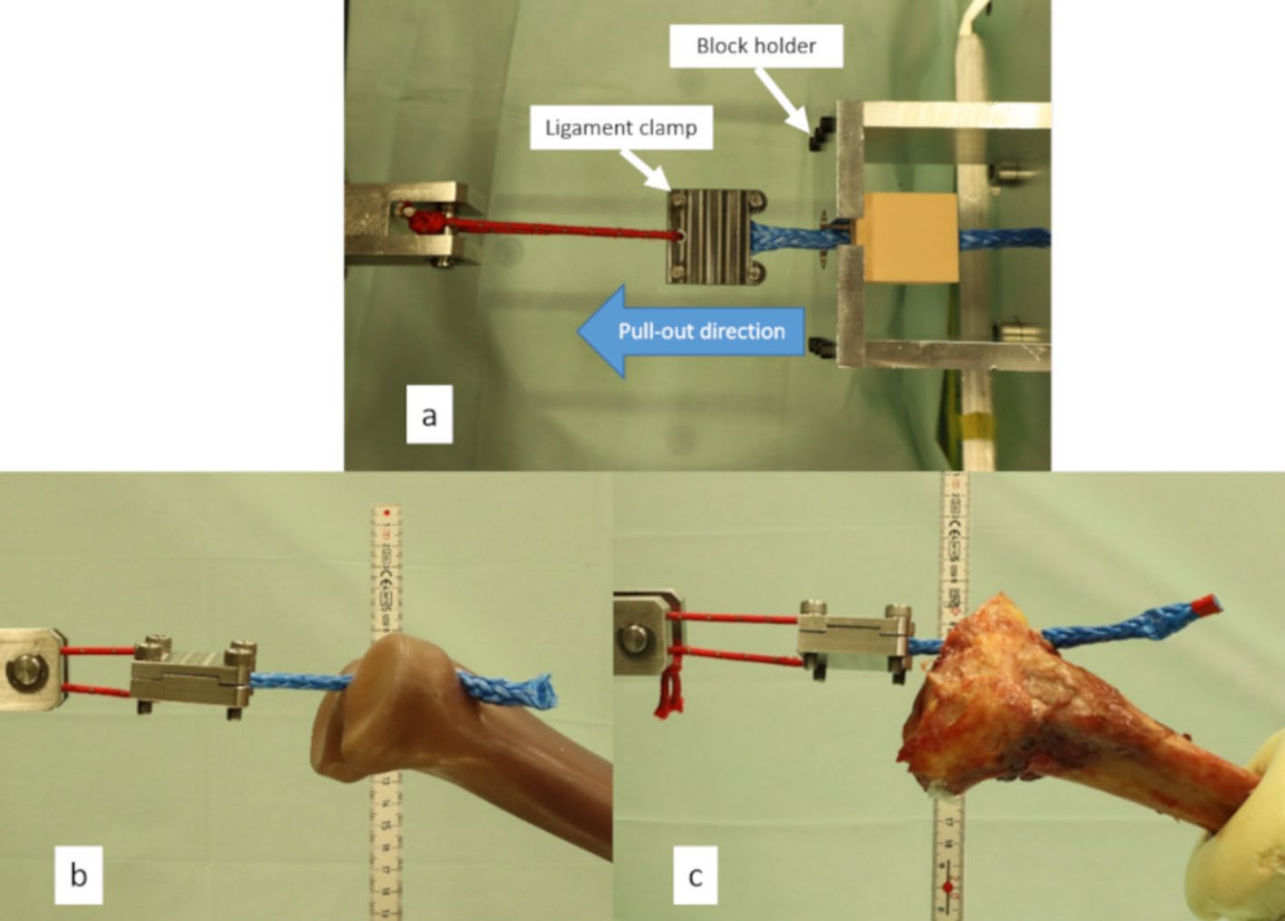
(**a**) Foam block test setup, (**b**) tibia model test setup, and (**c**) cadaveric tibia test setup.

The pull-out and dynamic tests were performed after the insertion tests. Figure 3a shows the test setup: the foam block (with an IS inserted) was placed into a block holder. The tibia model and cadaveric knee test setup are shown in Fig. 3b,c, respectively.

The pull-out test was performed at 6 mm/min and stopped when the machine reached a controlled maximum displacement of 78 mm. The pull-out force was obtained from the force–displacement curve determined by the maximum force achieved before the mode of failure occurred. As a mode of failure, any potential ligament material failure, slippage of the ligament material, or screw failure (loosening or fracture of the screw) was observed. The recorded pull-out force of each repetition was analyzed to obtain the average pull-out force of a specific test setup. The illustration of the load curve can be found in the supplementary information S1: the force reached its peak and decreased when a mode of failure occurred. At the end of the tests, the ISs were removed to measure the narrowest (minor) and widest (major) diameters of the tunnel at the insertion site using a digital Vernier calliper (Accud Co., Ltd, SuZhou, China). The narrow-wide ratio (aspect ratio) was calculated to represent the tunnel widening pattern (i.e., circular or elliptical tunnel). The dynamic test was performed at a frequency of 1 Hz for 1000 cycles with a load from 0 N to 200 N. Preconditioning of ten cycles at 25 N was performed in all tests with the porcine tendon. After the dynamic test, a pull-out test was performed. After the test, each bone material was cut longitudinal to the tunnel to observe the interaction between the bone and the screw. The screw was visually inspected for any damage.

### Statistics

Descriptive statistics were performed using mean and standard deviation (SD). Specimens were analyzed with a two-tailed t-test for independent samples. Statistical significance was set at P smaller than 0.05.

### Ethical statement

This study did not involve any live vertebrates and/or higher invertebrates, and therefore, ARRIVE guidelines is not applicable. An ethic votum from the ethic commission is not necessary, because all included specimens of the investigation are taken from persons who are included in the body donor program of the university hospital of Aachen (Uniklinik RWTH Aachen, Germany). Each person of this program signed a personal testament for using the body for medical education and medical investigations. Additionally, the authors also confirm that the experiment was performed in accordance with the regulations to obtain, experiment, and disposal of all cadavers.

## Results

**Figure 4:**
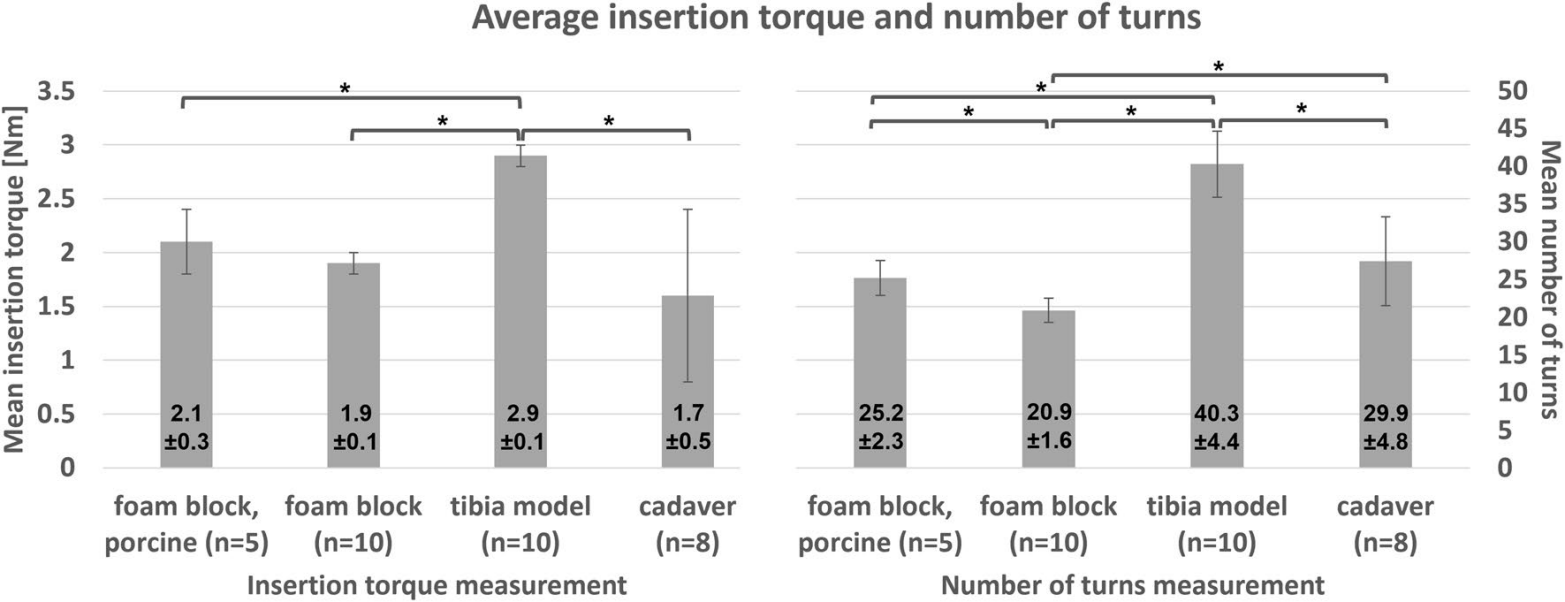
Average insertion torque and number of turns to complete the IS insertion (*P < 0.05).

A total of 36 ISs were used across the three materials. Overall, the average initial ligament tension before insertion was 40.6 ± 5.0 N. Figure 4 shows the average insertion torque and the number of turns required for complete screw insertion of all setups (foam block-porcine tendon; foam block-rope; tibia model-rope; and cadaver-rope). The comparing the average insertion torque between each setup found that the foam block-porcine tendon, foam block-rope, and cadaver-rope setup was not significantly different (P = 0.1, P = 0.2, and P = 0.4, respectively). However, the average insertion torque between the tibia model-nylon rope to the foam block-porcine tendon, foam block-rope, and cadaveric bone-rope setup was significantly different (P < 0.001) as the tibia model setup had higher insertion torque than the other. During screw insertion in the tibia model, the cortical layer and the nylon rope were strongly compressed, resulting in insertion torque going beyond the torque screwdriver limit. In this instance, we continued with a regular screwdriver until less torque was required, and measured the torque of the last few turns. The analysis of the tightening torque curve for the foam block and cadaveric tibiae with the nylon rope showed that the insertion torque increases once the IS starts advancing in the tunnel until it reaches the highest torque at the final turns. In the tibia model with nylon rope, at the beginning of the screw progression, the insertion torque exceeded 3 Nm and decreased towards the end, with the final insertion torque measured at less than 3 Nm. The number of turns required to insert the screw varied between the test setups. The comparison between all setups shows a significant difference (P < 0.001) except foam block-porcine tendon and cadaver-rope setup showed no significant difference (P = 0.1) between them.

**Figure 5:**
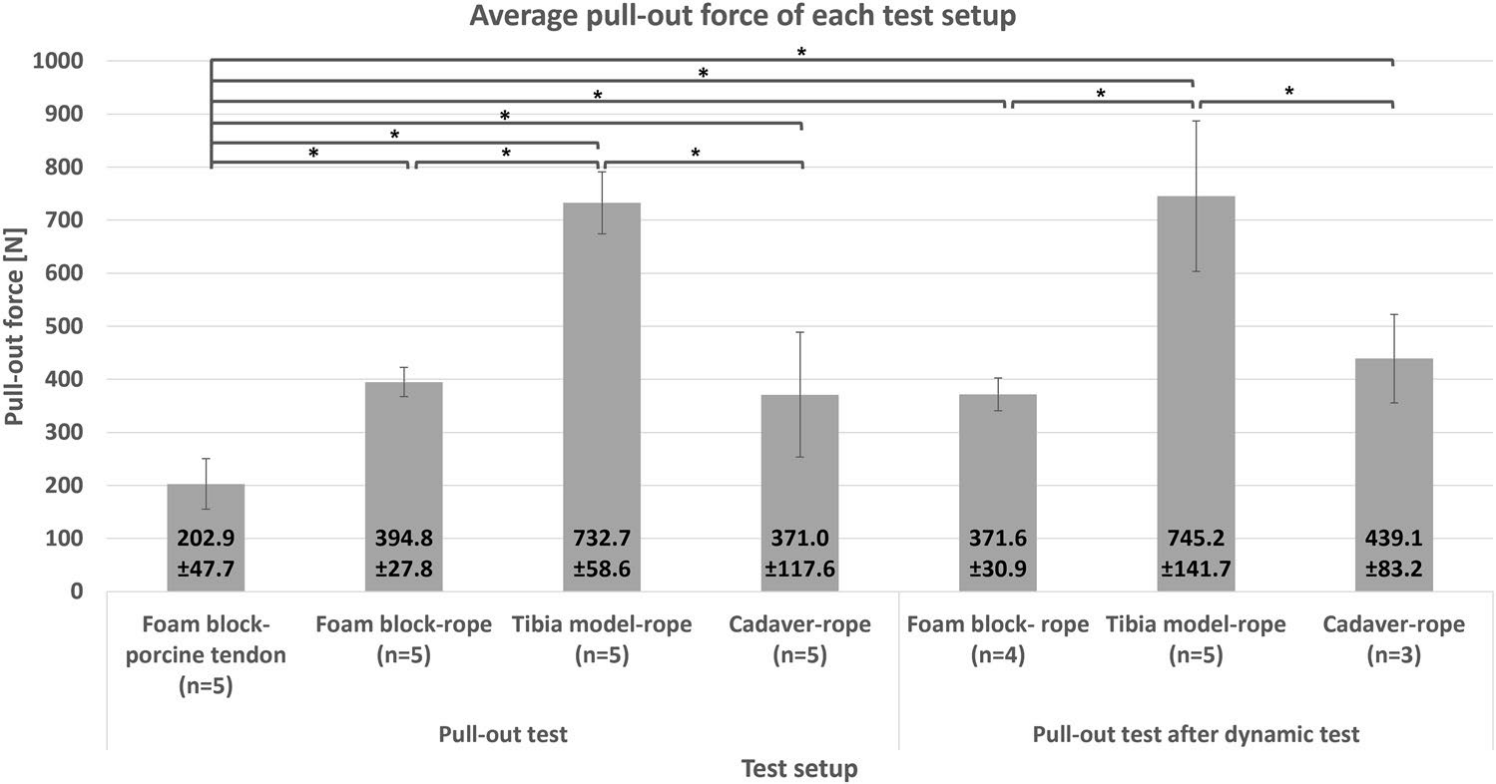
Average pull-out force for each test setup (*P < 0.05).

Figure 5 shows the average pull-out force for each test setup. The foam block-porcine tendon setup is significant different to all other setups (P < 0.001). There was no significant difference in the pull-out force when compared the pull-out test and pull-out after the dynamic test between the same bone material (P = 0.3, P = 0.9, and P = 0.4 for foam block, tibia model, and cadaveric tibiae, respectively). Examining the difference between the bone materials, the foam block-rope and cadaveric tibiae-rope setup showed no significant difference for both the pull-out and pull-out after the dynamic test (P = 0.3 and P = 0.1). In both the pull-out test and pull-out after the dynamic test, the tibia model-rope compared to the foam block-rope and cadaveric bone-rope demonstrated significant differences (P < 0.001, P < 0.001, P = 0.001, and P = 0.01, respectively).

In one dynamic test of foam block, the nylon rope slipped at 524 cycles. Therefore, only four pull-out force results were used for this combination (n = 4). In the cadaver test setting, only three of six pull-out tests could be used after the dynamic test (n = 3) for the statistical analysis, as some specimens were severely osteoporotic (Fig. 8d). The three failed cadavers show the following outcome: (1) insertion torque of 3.0 Nm, 0.2 Nm, and 0.3 Nm, respectively; (2) number of turns at 22, 20, and 20 turns, respectively; and (3) the pull-out force was not representative. Moreover, high insertion torque and number of turns in the tibia model setup were caused by the model structure, which will be later discussed.

**Figure 6:**
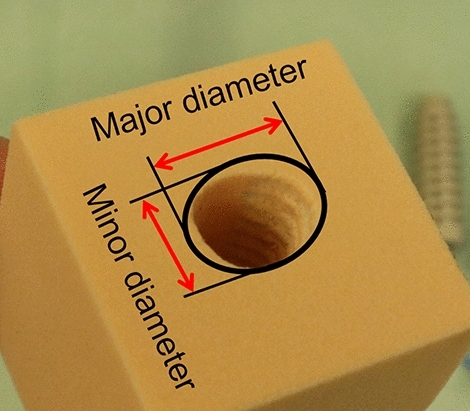
Measurement of tunnel widening (aspect ratio) at the insertion point.

**Figure 7:**
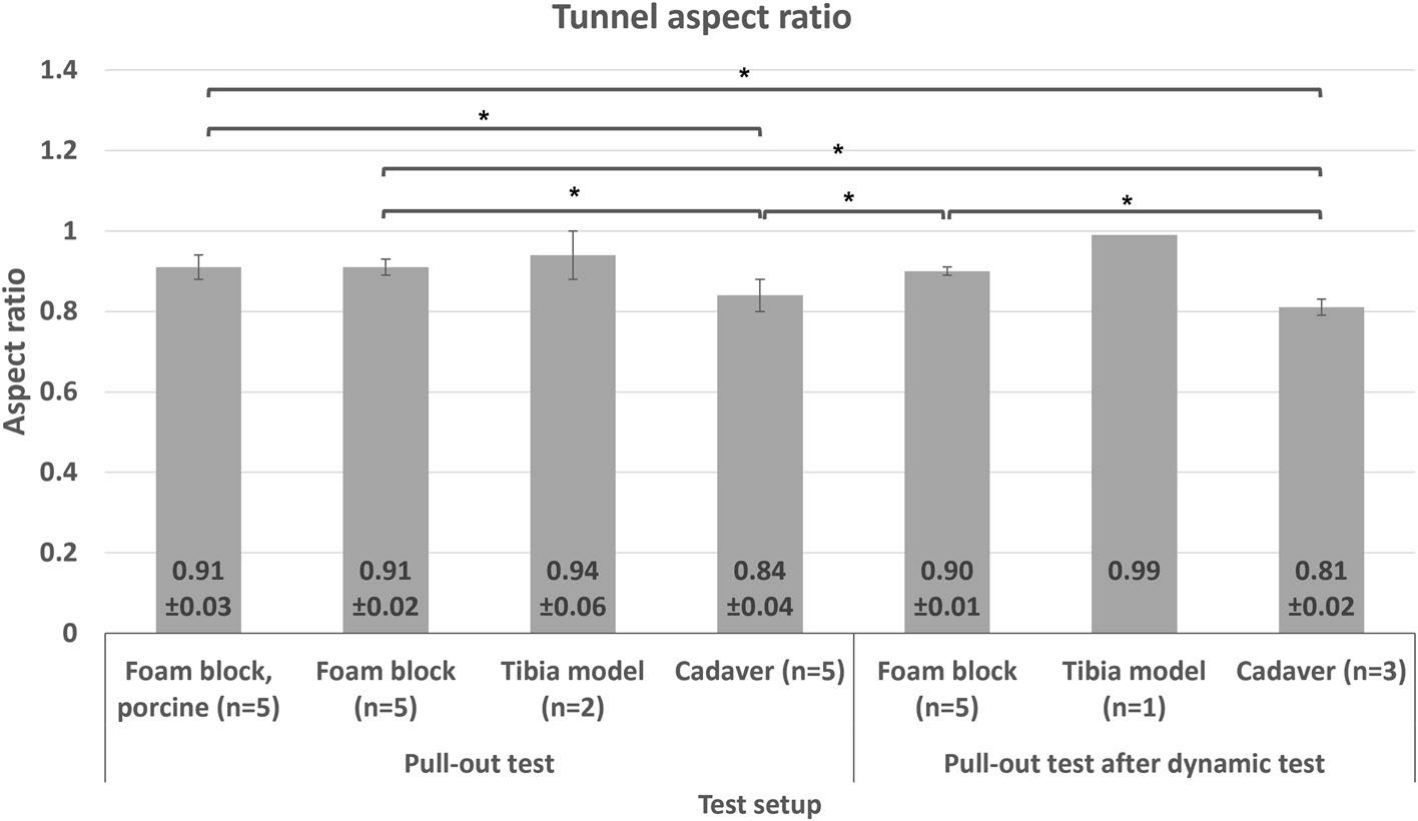
Aspect ratio of tunnel widening (*P < 0.05) without statistical analysis of the tibia model setup.

Figure 6 shows an example of tunnel widening in an elliptical pattern which major-minor diameter was measured. The information regarding tunnel size before and after the test and the tunnel widening pattern can be found in the supplementary information S1. Figure 7 shows the tunnel aspect ratio of all test setups. The results show no significant difference comparing the porcine tendon and nylon rope in a pull-out and dynamic test using a foam block (P = 0.8, P = 0.7, respectively). The comparison of the tunnel aspect ratio of the pull-out and dynamic test using foam block and cadaver shows no statistically significant difference (P = 0.2, and P = 0.2, respectively). The comparison between all foam block setups and cadaver setups is significantly different (P < 0.05). However, in the tibia model tests, only two tunnel widenings in the pull-out test (n = 2) and one tunnel widening in the pull-out after the dynamic test (n = 1) were measured because the interference screw could not be removed from the tibia. Given the small sample data in the tibia models, the data on tunnel widening were not statistically analyzed. From the observation of the experiment, the mode of failure by slippage of the ligament material. In all tests, all screws were placed in the same position, and never showed any sign of failure such as screw fracture or loosening.

**Figure 8:**
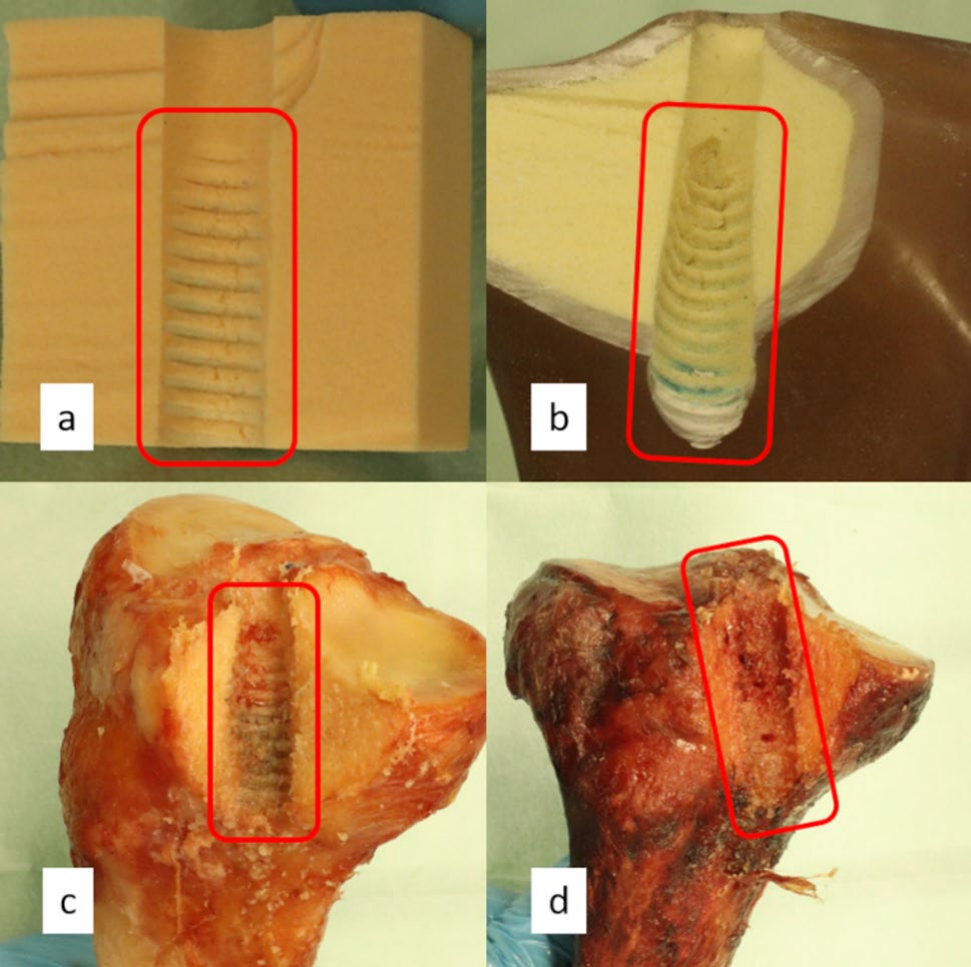
Examples of the interaction between the interference screw in (**a**) foam block, (**b**) tibia model, (**c**) healthy cadaveric tibia, and (**d**) osteoporotic cadaveric tibia.

Figure 8 shows examples of the interaction between the ISs and the insertion materials. Figure 8a–c shows the screw progression to the bone material from the insertion point through the length of the screw. On the other hand, in an osteoporotic cadaveric tibia, Fig. 8d, the trabecular bone structure could not withstand the load that occurred during the insertion. Therefore, the progression of the treads of the screw to the bone influenced the pull-out force resistance of the bone-screw setup.

## Discussion

Compared to polymer and permanent metal ISs, magnesium ISs offer the advantages of being bioabsorbable and strong, preventing imaging artefacts, and allowing less complicated revision procedures^24,25^. This study tested the performance of one magnesium IS design. To achieve the aim of investigating the insertion and fixation performance of the screw, the insertion tests, pull-out tests, and dynamic tests were performed to measure the insertion torque, number of turns, pull-out force and widening ratio in several scenarios of bone materials.

The performance testing method for ISs approximated the guideline for the in vitro testing of the cruciate ligaments and ligament reconstruction^17^, and was adapted from the standard specification and test methods for metallic medical bone screws (ASTM F543). Fixation strength is determined via a pull-out test (load-to-failure test) and by a dynamic test. The pull-out test can be performed with a pulling rate ranging from 5 to 1000 mm/min^17^. The dynamic test is performed through 1000 cycles which can evaluate the initial response of the IS-graft construct^17^. The ligament should be preconditioned in a low cyclic-low load manner, for example, with 20 cycles ranging between 0 and 50 N^18^. Three materials are commonly employed for IS testing: polyurethane foam blocks, a Sawbones tibia model, and human cadaveric knees^3,4,19–22^. In addition, for the tibia model, the tunnel of 65° in the coronal and sagittal plane is a practical tibia tunnel range of 52°–75° and 64°–80° in the coronal and sagittal plane, respectively^26–28^.

In the insertion test, firstly, the initial ligament tension force before the insertion was recorded. In single bundle ACL reconstruction, an ACL tension between 20 N and 90 N is recommended to obtain better knee stability^29^. Hence, the average ACL tension force of 40.6 ± 5.0 N in the present investigation is within the recommended range. Secondly, the insertion torque indicates the work required by surgeons to insert the screw^4^. The insertion torque of less than 3 Nm lies within the normal insertion torque of ISs^26,27^ and is congruent to the literature, as the insertion torque remains less than 3 Nm throughout the experiment^14^. The different bone materials and ligament setups influence the insertion torque and the number of turns required for the IS insertion. The test results gave information on the insertion performance of the screw, and various materials may require a different amount of work. In this study, experimental consistency in a small amount of repetition (in various bone materials) was essential. Therefore, using one surgeon would have provided a more consistent outcome in the investigation. The number of turns only implies the amount of work of an individual surgeon. It is not a goal to achieve a certain number of turns during the actual operation. Obviously, further studies involving multiple surgeons can be conducted in the future.

In this study, the consultant surgeon remarked that, compared to the IS insertion in an actual operation, the IS insertion using healthy cadaveric tibiae gave the most realistic physical feedback/feeling followed by the foam block-nylon rope setup. In addition, the surgeon performed tunnel drilling in the cadaveric tibia, which should better reflect the clinical setting. In contrast, in the artificial tibia model-nylon rope setup, the high compression between the cortical layer of the tibia model and the nylon rope caused an unrealistic haptic insertion response.

Regarding the bone materials, the experimental issues with unexpectedly high insertion and pull-out force of the tibia model can be related to the construction of the tibia model itself. From the higher tunnel aspect ratio at the insertion point, the tibia has a more rounded shape compared to the more elliptical shape of the foam block and cadaver. One more point is when the IS reached inside the 17 PCF foam core, in the last few turns, the insertion lies within the 3 Nm again. This implies that a hard cortical layer influences the increase in the insertion torque beyond 3 Nm and decreased in the last few turns. This results from the material property and the thickness of the tibia model’s cortical layer, which responds differently in the foam block and the cadaver specimen. This produced an unrealistically high insertion torque (> 3 Nm) at the start and decreased when the screw advanced to the trabecular 17 PFC foam layer of the model to reach the final insertion torque of less than 3 Nm. However, with this finding, shows that this issue probably results from the nature of the tibia model, not from the IS itself. Moreover, the magnesium IS can withstand high compression forces during the insertion, and a higher than 3 Nm insertion torque.

ISs provide sufficient initial fixation. The ligament slippage occurred only to the ligament material while the screw remained well fixed to the bone. The use of nylon rope eliminates biological variability. In this way, screw performance and bone-screw interaction can be better characterized. Using the nylon rope resulted in a narrower SD than the porcine tendon, given more consistent material parameters of the rope. In an artificial tibia model, as a consequence of the cortical layer structure, a high pull-out force for both pull-out and pull-out after the dynamic test occurred. On the other hand, the foam block and cadaveric tibiae showed similar pull-out force, whereas the foam block had a narrower SD. There was no statistical difference between the foam block and cadaveric tibiae setup. After the dynamic test, the cadaveric tibiae pull-out force was slightly higher than the initial cadaver pull-out test. This is congruent with other studies^28^: the IS retains the same fixation ability after 1000 cyclic loads. The ACL graft force was estimated in situ between 30 and 450 N, with 150 N sufficient to undertake daily activities such as walking^28^. This initial fixation value may vary between studies according to the experimental setup, from 200 N to more than 1000 N^4,25,30^. However, the IS provides sufficient fixation strength in both single pull-out and dynamics loading. One additional observation of a study with a similar setup^14^ shows that the pull-out force in the porcine tendon setup is significantly different to the nylon rope setup.

The tunnel widening pattern shows the bone-screw-ligament interaction. The ratio of approximately 0.91 in all foam block setups implies that the screw compresses the ligament material to one side of the tunnel and the wall on the other side, producing an elliptical tunnel. The tunnel widening ratio in the cadaver tests was 0.84 and 0.81 for pull-out and pull-out after the dynamic test, respectively. The tunnel was narrower and longer at the end. Unfortunately, tunnel widening for the tibia model was not comparable given the non-realistic insertion pattern, and the screws were stuck under the cortical layer. It was possible to remove only three of ten screws from the tibia model. The tunnel widening ratio in the tibia model was measured from the cortical layer. The tunnel widening ratio warrants further investigation into the progression of the screw tread to the tunnel wall. The foam block setup showed consistent progression of the screw to the tunnel wall for the whole length of the screw. This means that, while the screw progressed into the bone on one side, the screw also compressed the ligament on the other side without ligament rotation in the tunnel. This showed good screw function in terms of bone-screw and graft-screw interaction. The artificial tibia model also showed a screw-bone interaction similar to what was evidenced in the bone block.

When observing the cut tunnel, the thickness of the cortical layer was evident from what was mentioned regarding the construction of the tibia model. The cortical layer of the tibia model is thicker and possibly harder (from the tunnel aspect ratio implication) than the cortical layer of the cadaveric tibia shown in Fig. 8a,c. However, the material properties of the cortical layer of the tibia model and cadaver were not investigated. Furthermore, the blue-colored mark from the nylon rope indicated higher compression in the cortical layer region where the blue-colored mark was not found in both foam block and cadaver. Overall, the use of tibia model in this study gave useful information on the strength of the magnesium IS. However, in the future, the utilization of the tibia model has to be carefully studied and planned to achieve a promising result.

The tunnel widening pattern of healthy and osteoporotic bones was similar but resulted in differences in the pull-out and dynamic test. This can be explained by observing the progression of ISs to the bone. The healthy bone showed screw tread progression to the bone morphological structure while the osteoporotic bone visually showed no progression of the screw to the bone (Fig. 8). The bone structure and the bone mineral density were not quantified, but visually the healthy bone is denser than the osteoporotic bone, likely resulting in better IS-bone interaction. Greater bone mineral density influences the initial fixation of the screw^4,17,28,31^.

The three bone materials used in this study to investigate the performance of the IS in various settings are all commonly used in biomechanical testing. In this article, the tibia model may not suit this experimental setup but shows a promising outcome when using extracortical graft fixation^21^. The cadaveric and foam block can be used interchangeably for insertion torque and pull-out force investigation. The mechanical properties and any pathological condition of the bone may also influence the outcome, with an inconsistent result or a failure test as shown in the test with osteoporotic bones.

Our study presents some limitations. First, for the assessment of the IS insertion, the experimental setup did not include any device to measure the axial force applied through the screwdriver. Therefore, axial force should be measured in addition to the measurement of the torque and number of turns. By measuring the axial force, torque, and the number of turns, the work required to implement the IS can be better evaluated. Second, in the insertion test, only the maximum insertion torque and the number of turns (as an attempt to rotate) were recorded. To improve the assessment, the insertion torque and the rotation angle could be recorded at each screw turn. In this way, the insertion torque-turn angle-number of turns curve can be investigated. These results are only applicable to screws with a diameter of 9 mm and length of 30 mm. Future studies should investigate different screw dimensions. Lastly, the testing machine reached its limit in the pull-out test at 6 mm/min. Even though the pull-out rate at 5 mm/min was suggested regarding the ASTM F543, the pull-out rate between 2.5 and 7.5 mm/min does not significantly affect the outcome^32^.

## Conclusions

Magnesium ISs show suitable insertion and fixation performance in all test setups without any indication of screw damage. In the insertion test, magnesium ISs show good workability in terms of the amount of work required for IS insertion (maximum torque and number of turns). The use of foam blocks and cadaveric tibiae also give realistic feedback on IS insertion. Both foam blocks and cadaveric tibiae in combination with nylon rope are also suitable for this experimental approach. The major disadvantage of cadaveric tibiae is that they are biological material; hence, the osteoporotic bone may lead to test failure. In all experimental settings, including high insertion torque in the tibia model, the magnesium ISs demonstrated good material strength and good bone-screw-ligament interaction. The bone progression and tunnel widening pattern show the ability of the magnesium ISs to compress the ligament providing suitable initial graft fixation. Further investigations on the technical and mechanical properties of magnesium ISs should be performed.

## Supplementary Information

Supplementary Information.

## Data Availability

All data are available under reasonable request to Nad Siroros (nsiroros@ukaachen.de).
