# Supplementary material for: Mechanical properties of a bioabsorbable magnesium interference screw for anterior cruciate ligament reconstruction in various testing bone materials
Source: Sci Rep. 2023 Jul 31;13:12342. doi: 10.1038/s41598-023-39513-8 (PMC10390578; doi:10.1038/s41598-023-39513-8)
Supplement: Supplementary file 1 — Supplementary Information. [file 41598_2023_39513_MOESM1_ESM.pdf]

Supplementary Information

Mechanical properties of a bioabsorbable magnesium interference screw for anterior cruciate ligament reconstruction in various testing bone materials

Nad Siroros [1], Ricarda Merfort [1], Yu Liu [1], Maximilian Praster [1], Filippo Migliorini [1,*], Nicola Maffulli [2,3,4], Roman Michalik [1], Frank Hildebrand [1], and Jörg Eschweiler [1]

* Correspondence: migliorini.md@gmail.com

Supplementary Figures

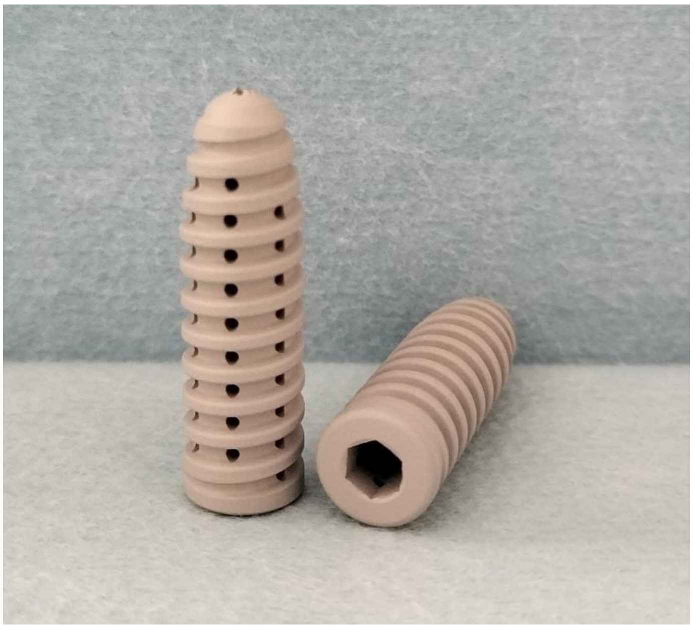

Figure 1. A bioabsorbable magnesium interference screw for ACL fixation was used in the study. ´

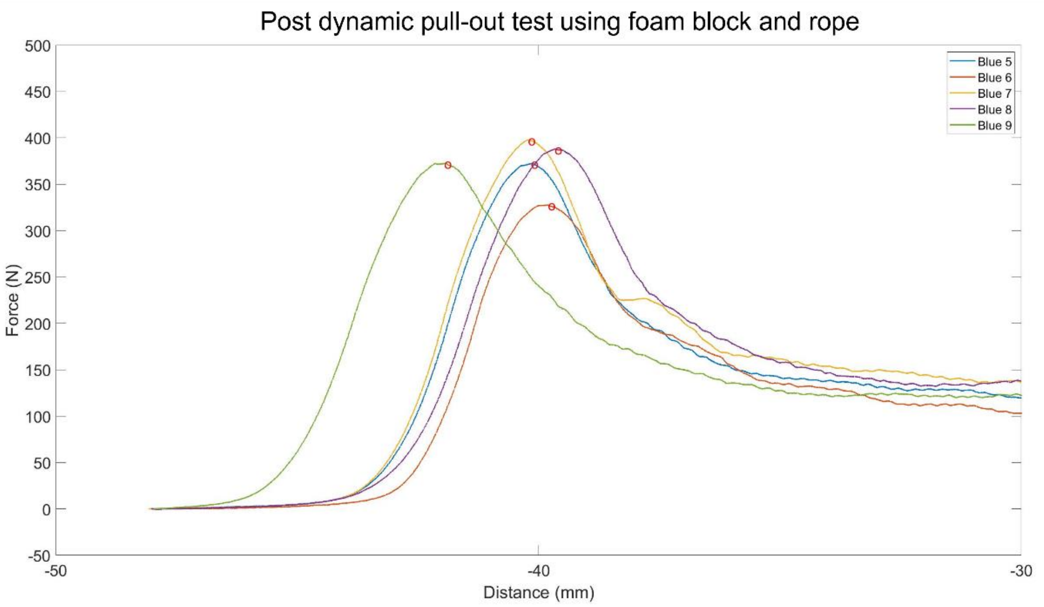

Figure 2. An illustration of the load curve to determine the pull-out force.

Supplementary Table

Table 1. Average insertion torque, the number of turns for all test setups.

Test setup	Sample size	Insertion torque [Nm](Nm)	No. of turns
foam block, porcine	n=5	2.06 ±0.32	25.20 ±2.28
foam block	n=10	1.88 ±0.09	20.90 ±1.60
tibia model	n=10	2.92 ±0.09	40.30 ± 4.42
cadaver	n=11	1.57 ±0.85	27.36 ±5.92

Table 2. Average tunnel size before and after the test and the tunnel widening pattern.

Test setup	Sample size	Tunnel diameter before insertion (mm)	Tunnel diameter after insertion		
			Minor (mm)	Major (mm)	Aspect ratio
Pull-out, foam block, porcine	n=5	9.02 ±0.01	9.64 ±0.34	10.62 ±0.19	0.91 ±0.03
Pull-out, foam block	n=5	10.02 ±0.03	10.27 ±0.08	11.26 ±0.21	0.91 ± 0.02
Pull-out, tibia model	n=2	10.04 ±0.01	10.06 ±0.17	10.69 ±0.55	0.94 ± 0.06
Pull-out, cadaver	n=5	8.79 ±0.18	9.42 ±0.16	11.24 ±0.65	0.84 ±0.04
Dynamic, foam block	n=5	10.04 ±0.02	10.18 ± 0.14	11.30 ±0.07	0.90 ± 0.01
Dynamic, tibia model	n=1	10.00 ±0.09	10.13	10.27	0.99
Dynamic, cadaver	n=3	8.62 ±0.04	9.20 ±0.06	11.43 ±0.31	0.81 ± 0.02
